# Complete Genome Sequence of *Caulobacter crescentus* Siphophage Sansa

**DOI:** 10.1128/genomeA.01131-15

**Published:** 2015-10-08

**Authors:** Leonardo Vara, Ashley A. Kane, Jesse L. Cahill, Eric S. Rasche, Gabriel F. Kuty Everett

**Affiliations:** Center for Phage Technology, Texas A&M University, College Station, Texas, USA

## Abstract

*Caulobacter crescentus* is a Gram-negative dimorphic model organism used to study cell differentiation. Siphophage Sansa is a newly isolated siphophage with an icosahedral capsid that infects *C. crescentus*. Sansa shares no sequence similarity to other phages deposited in GenBank. Here, we describe its genome sequence and general features.

## GENOME ANNOUNCEMENT

*Caulobacter crescentus* is an aquatic oligotrophic bacterium capable of growing in low-nutrient environments (1). *C. crescentus* forms two types of cells, stalk and swarmer cells, and is a model organism for cell differentiation (2). Bacteriophage Sansa was isolated on *C. crescentus* strain CB15. Bacteriophages have long been a tool used in *C. crescentus* research (3), and Sansa may be useful to further study this model organism.

Bacteriophage Sansa was isolated from a water sample collected in Houston, TX. Phage DNA was sequenced using 454 pyrosequencing at the Emory GRA Genome Center (Emory University, GA). Trimmed FLX Titanium reads were assembled to a single contig of circular assembly at 24.9-fold coverage using the Newbler assembler, version 2.5.3 (454 Life Sciences), with the default settings. The contig was confirmed to be complete by PCR using primers that face the upstream and downstream ends of the contig. Products from the PCR amplification of the junctions of concatemeric molecules were sequenced by Sanger sequencing (Eton Bioscience, San Diego, CA). Genes were predicted using GeneMarkS (4) and corrected using software tools available on the Center for Phage Technology (CPT) Galaxy instance (https://cpt.tamu.edu/galaxy-public/). Morphology was determined using transmission electron microscopy performed at the Texas A&M University Microscopy and Imaging Center.

Sansa is a siphophage with a double-stranded DNA (dsDNA) unit genome of 78,170 bp, a coding density of 95.4%, and a G+C content of 65.8%. A 2,127-bp repeat was determined using the PAUSE method on raw sequencing data (https://cpt.tamu.edu/pause/). The repeat contains 7 coding sequences resulting in a final packaged genome of 80,297 bp with 122 coding sequences. Of the 122 coding sequences, 115 are unique (does not include duplicate coding sequences in the terminal repeat). Thirty-six coding sequences are homologous to proteins of known function, as determined by BLASTp and InterProScan (5, 6). Sansa shows no similarity to other phages in the GenBank database, as determined by BLASTn analysis (7).

Genes encoding proteins involved in DNA replication and recombination, DNA packaging, morphogenesis, and lysis were identified. Genes encoding DNA replication and recombination proteins include those for primase, polymerase III, several DNA-binding proteins, single-stranded DNA (ssDNA)-annealing protein, nucleases, and helicase. The genes encoding the ssDNA-binding protein and the DNA primase share 77% and 76% nucleotide sequence identity with the corresponding regions on the CB15 and K31 genomes, respectively. DNA packaging genes encoding the portal protein, large and small terminase, and a DNA packaging protein were annotated. The DNA packaging protein is in the Terminase_3 superfamily (accession no. cl12054), as determined by CD Search (8). The structural proteins identified include the major capsid, tail, tailspike, and tape measure proteins. Additional structural genes were not identified, likely due to a lack of sequence similarity to other phages. Sansa encodes several methylase proteins, including an adenine-specific DNA methyltransferase. A lysis cassette was identified that contains a putative holin/antiholin pair, endolysin, and partially embedded inner- and outer-membrane spanins (9, 10).

### Nucleotide sequence accession number.

The genome sequence of phage Sansa was contributed as accession no. KT001913 to GenBank.
